# Molecular Cloning, Expression, and Functional Analysis of Glycosyltransferase (TbUGGT) Gene from *Trapa bispinosa* Roxb.

**DOI:** 10.3390/molecules27238374

**Published:** 2022-11-30

**Authors:** Shijie Ye, Dongjie Yin, Xiaoyan Sun, Qinyi Chen, Ting Min, Hongxun Wang, Limei Wang

**Affiliations:** 1 College of Life Science and Technology, Wuhan Polytechnic University, Wuhan 430023, China; 2 College of Food Science and Engineering, Wuhan Polytechnic University, Wuhan 430023, China

**Keywords:** *Trapa bispinosa* Roxb., TbUGGT, molecular cloning, expression analysis

## Abstract

*Trapa bispinosa* Roxb. is an economical crop for medicine and food. Its roots, stems, leaves, and pulp have medicinal applications, and its shell is rich in active ingredients and is considered to have a high medicinal value. One of the main functional components of the *Trapa bispinosa* Roxb. shell is 1-galloyl-beta-D-glucose (βG), which can be used in medical treatment and is also an essential substrate for synthesizing the anticancer drug beta-penta-o-Galloyl-glucosen (PGG). Furthermore, gallate 1-beta-glucosyltransferase (EC 2.4.1.136) has been found to catalyze gallic acid (GA) and uridine diphosphate glucose (UDPG) to synthesize βG. In our previous study, significant differences in βG content were observed in different tissues of *Trapa bispinosa* Roxb. In this study, *Trapa bispinosa* Roxb. was used to clone 1500 bp of the UGGT gene, which was named TbUGGT, to encode 499 amino acids. According to the specificity of the endogenous expression of foreign genes in *Escherichia coli*, the adaptation codon of the cloned original genes was optimized for improved expression. Bioinformatic and phylogenetic tree analyses revealed the high homology of TbUGGT with squalene synthases from other plants. The TbUGGT gene was constructed into a PET-28a expression vector and then transferred into *Escherichia coli* Transsetta (DE3) for expression. The recombinant protein had a molecular weight of 55 kDa and was detected using SDS-PAGE. The proteins were purified using multiple fermentation cultures to simulate the intracellular environment, and a substrate was added for in vitro reaction. After the enzymatic reaction, the levels of βG in the product were analyzed using HPLC and LC-MS, indicating the catalytic activity of TbUGGT. The cloning and functional analysis of TbUGGT may lay the foundation for further study on the complete synthesis of βG in *E. coli*.

## 1. Introduction

*Trapa bispinosa* Roxb. is an annual herbaceous floating plant belonging to Myrtle’s *Trapa bispinosa* family and is mainly distributed in tropical and temperate regions [1,2]. Ripe *Trapa bispinosa* Roxb. has a hard deep red shell and creamy white flesh with a sweet taste. It can be used to treat common diseases, such as gastric ulcers, esophageal cancer, and dysentery [3]. The fruit hulls are rich in phenols and flavonoids and have been extensively studied. The extracts from *Trapa bispinosa* Roxb. shell contain many phenolic compounds, such as gallic acid, caffeic acid, naringin, and 1,2,3,4,6-pentagalacyl-β-D-glucose [4,5,6]. These phenolic compounds have specific physiological antioxidant, anti-inflammatory, and anticancer properties [7,8,9,10]. *Trapa bispinosa* Roxb. chestnut is native to Europe and Asia but is only cultivated in China and India [11]. The plant exists in most water bodies in China but is considered one of the aquatic specialties of the Hubei Province and has high economic value.

The compound 1-galloyl-beta-D-glucose (βG) exists in plants such as oak leaves, *Trapa bispinosa* Roxb., and pomegranate and possesses a variety of pharmacological activities. Studies have shown that βG is a noncytotoxic and selective AKR1B1 inhibitor of aldose reductase, which can protect against oxidative stress and treat secondary complications of diabetes [12]. In a previous study, the protective properties of βG and its mitochondrial antioxidative mechanism reduced the effects of oxidative stress in glaucoma [13]. In Raw 267.4 macrophages, βG prevented LPS-induced activation of JNK and p38 and lowered ROS levels [14]. APRE-19 cells pretreated with βG demonstrated decreased apoptosis induced by retinal microglia [15]. Furthermore, βG inhibited the activation of the NLRP3 and TLR4/NF-κB pathways and decreased the expression of pro-inflammatory cytokines, protecting against LPS-induced sepsis in mice and reducing organ toxicity [16]. The substance has a wide range of applications in clinical practice, but βG is primarily produced via plant extraction, which does not yield high quantities. Therefore, chemical synthesis methods are also being developed but are in opposition to the green concept of modern production. At the same time, biosynthesis has become a popular method for material acquisition. In this study, we aim to develop a βG production method using biosynthesis to meet the medical application demand.

Glycosyltransferase (EC 2.4.x.y) is an enzyme that can transfer activated glycol groups to other small-molecule compounds to complete glycosylation reactions [17]. UGT is a soluble enzyme in plants, with UDPG being the leading sugar donor [18]. According to the different substrate small molecules, the UGT family can be divided into UGGT with gallic acid as the substrate and UDP-glucose with flavonoids as the substrate, such as flavonoid 3-glucosyltransferase (UFGT) [19], etc. UGGT plays an essential role in catalyzing the formation of βG in plant tannin biosynthesis.

**Figure 1:**
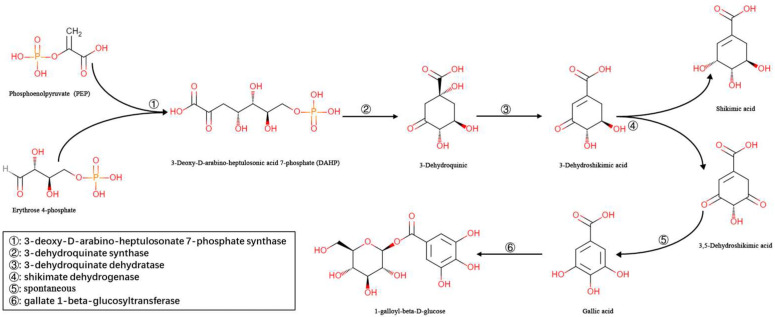
Biosynthesis of βG in some higher plants.

U-glutamyl transpeptidase (UGGT) catalyzes the synthesis of βG from gallic acid (GA) and uridine diphosphate glucose (UDPG) [20]. In some higher plants, GA is synthesized via the shikimic acid pathway and converts 3-dehydroshikimic acid to 3,5-dehydroshikimic acid through aroDE enzymes. In addition, 3,5-dehydroshikimic acid can be spontaneously converted to GA via enolization [21,22,23]. Figure 1 displays the chemical reaction scheme for βG synthesis.

Our previous research study identified this substance in *Trapa bispinosa* Roxb., with varying amounts in different tissues [24]. So far, most of the studies on *Trapa bispinosa* Roxb. have focused on the separation and pharmacological effects of active monomer compounds. However, the specific biosynthetic mechanism of the active components of *Trapa bispinosa* Roxb. has rarely been explored. Currently, the secondary metabolites identified in *Trapa bispinosa* Roxb. contain multiple types of galacyl glucose, using the biosynthetic βG of galacyl glucose as the substrate. However, the gene for its synthesis has not been reported.

The TbUGGT gene sequence was obtained with gene annotation and screening using transcriptomics technology. After codon optimization, the recombinant expression vector was constructed and expressed in *Escherichia coli,* and the enzyme activity was determined. This study lays the foundation for future research on the complete synthesis of βG in *Escherichia coli*.

## 2. Results

### 2.1. TbUGGT Gene Cloning and Sequence Analysis

**Figure 2:**
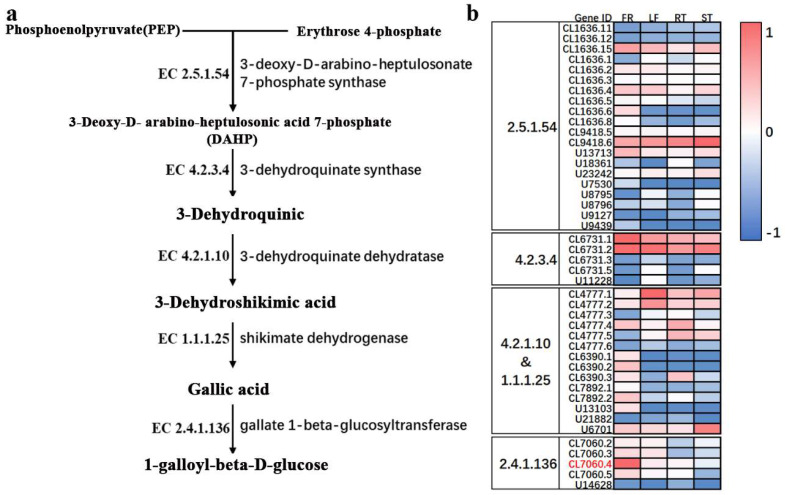
βG biosynthesis pathway (**a**) and the expression of related genes (**b**).

The βG biosynthesis pathway starts from phosphoenolpyruvate and D-erythritose 4-phosphate (Figure 2a). Combined with the transcriptome data of *Trapa bispinosa* Roxb., the Unigene expression belonging to this pathway detected using RNA-Seq was analyzed and displayed with a heat map (Figure 2b). A total of 44 Unigenes belonging to this pathway were identified using the transcriptome, which was involved in four genes of the pathway, namely, 3-deoxy-D-Arabino-Heptulosonate 7-phosphate synthase (EC 2.5.1.54), 3-dehydroquinate synthase (EC4.2.3.4), 3-Dehydroquinate dehydratase/Shikimate dehydrogenase (EC 4.2.1.10/EC 1.1.1.25), and gallate 1-beta-glucosyltransferase (EC 2.4.1.136). The primary glycosyltransferase gene TbUGGT (CL7060.4) was obtained.

The *Trapa bispinosa* Roxb. transferase gene TbUGGT was amplified with PCR using specific primers and the preferred codon optimization of *Escherichia coli*. The gene had a total length of 1500 bp, encoding 499 amino acids. The molecular weight of the protein sequence predicted using ProtParam was about 55.8 kDa and was an unstable hydrophilic protein.

**Figure 3:**
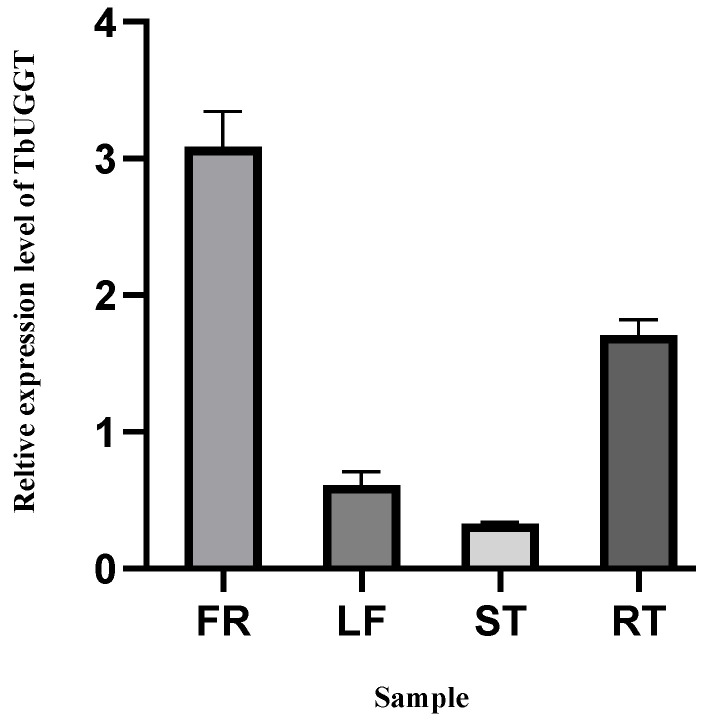
Differential expression of TbUGGT:TbUGGT in different growth sites, including shell (FR), leaf (LR), stem (ST), and root (RT).

qRT-PCR was used to detect the expression pattern of TbUGGT and identify the expression of the TbUGGT gene in different tissues of *Trapa bispinosa* Roxb., namely, shell (FR), leaf (LR), stem (ST), and root (RT) (Figure 3). Apparent differences in transcription levels were observed in different parts, the highest being shell (FR) expression, and the root (RT) expression being the second. In contrast, the stem (ST) and leaf (LR) yielded relatively low expressions.

According to the conservative structural domain analysis (Figure S1 Supplementary Material), CD Search predicted that the TbUGGT protein belonged to the glycosyltransferase_GTB-Type (PLN02555) superfamily with a domain range of 1aa–473aa (Figure S1a). ScanProsite predicted that the protein belonged to the UDP Glycosyltransferases superfamily with a domain range of 343aa–386aa (Figure S1b), while Pfam predicted that the protein belonged to the UDPGT family. The domain range was 238aa–423aa (Figure S1c). ProtScale indicated that the TbUGGT protein was hydrophilic (Figure S2). The signal peptide prediction showed no signal peptides in this protein, and the probability of amino acids in each point appearing outside the membrane was close to 1, demonstrating a low probability to appear in the transmembrane region. Therefore, this protein was not a membrane or secreted protein (Figure S3).

### 2.2. Structure and Phylogenetic Analyses

**Figure 4:**
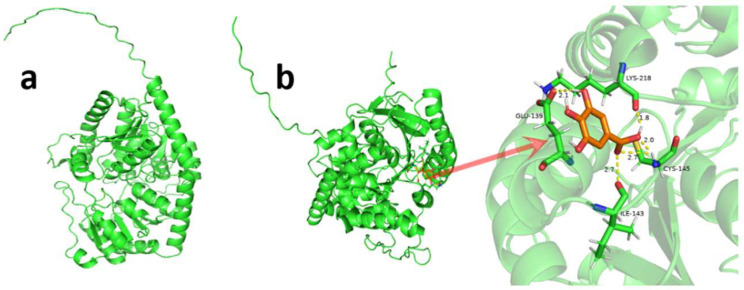
AlphaFold2 predicted TbUGGT protein structure model (**a**) and PyMOL software molecular docking results (**b**).

SOPMA showed that the secondary structure of the TbUGGT protein sequence contained α helices (blue), extended chains (red), β rotations (green), and random curls (purple), accounting for 40.48%, 14.23%, 4.21%, and 41.08%, respectively (Figure S4). The protein structure of TbUGGT was predicted using AlphaFold2, in which the model pLDDT was as high as 91.8. (Figure 4a). pLDDT ≥ 90 means that the residue has very high model confidence, which the model can use for later molecular docking analyses. The homology modeling structure was analyzed using PyMOL software (Figure 4b). The docking results between the protein model and the substrate GA molecule showed a binding energy of −6.3. A smaller binding energy indicated a tighter binding between the receptor and the ligand. Visualization revealed that the binding sites were mainly concentrated in Glu at position 139, in Ile at position 143, in Cys at position 145, and in Lys at position 218.

**Figure 5:**
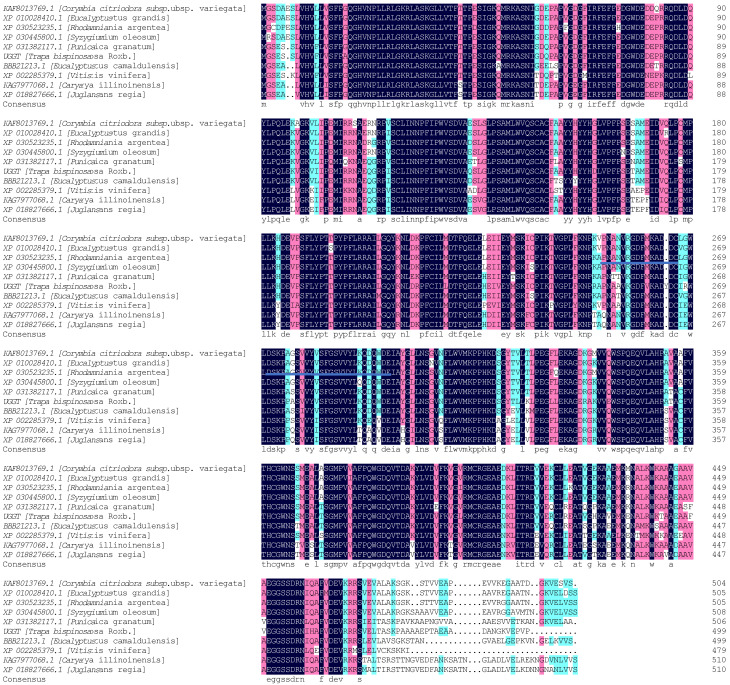
Comparison of TbUGGT amino acid sequences obtained from GenBank. The species, protein names, and GenBank accession number of the aligned sequences are as follows: *Corymbia Citriodora* subsp. variegata (KAF8013769.1); *Eucalyptustus grandis* (XP 01 0028410.1); *Rhodamniania argentea* (XP 030523235.1); *Syzygiumium oleosum* (XP 030445800.1); *Punicaica granatum* (XP 031382117.1); *Eucalyptus Camaldulensis* (BBB21213.1); *Vitis Vinifera* (XP 002285379.1); *Caryaya illinoinensis* (KAG7977068.1); *Juglansins regia* (XP 018827666.1).

In the homology analysis of the TbUGGT protein sequence, the compared species included *Punica granatum*, *Syzygium oleosum*, *Eucalyptus Grandis*, *Corymbia Citriodora* subsp. Variegata, *Eucalyptus Camaldulensis*, *Rhodamnia argentea*, *Juglans regia*, *Carya illinoinensis*, and *Vitis Vinifera*. The results showed a similarity of 87.60% between the protein and the compared sequence (Figure 5). About 44 amino acid residues in the blue underlined part of Figure 5 correspond to the conservative domain PSPG of glycosyltransferase [25], which is the binding region of glycosyl donors, suggesting that the cloned gene was the UDP glycosyltransferase gene.

**Figure 6:**
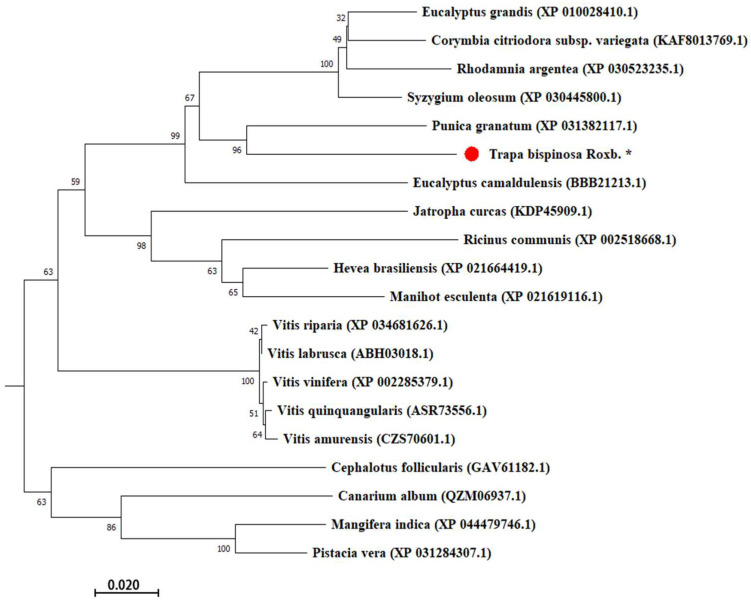
Phylogenetic tree of TbUGGT from various species. • *Trapa bispinosa* Roxb.*: The target gene TbUGGT in this study. The species, protein names, and GenBank accession number are *Eucalyptus Grandis* (XP010028410.1), *Corymbia Citriodora* subsp. Variegata (KAF8013769.1), *Vitis amurensis* (CZS70601.1), *Syzygium oleosum* (XP030445800.1), *Punica granatum* (XP031382117.1), *Eucalyptus Camaldulensis* (BBB21213.1), *Jatropha curcas* (KDP45909.1), *Ricinus communis* (XP002518668.1), *Hevea brasiliensis* (XP021664419.1), *Manihot esculenta* (XP 021619116.1), *Vitis Riparia* (XP 034681626.1), *Vitis labrusca* (ABH03018.1), *Vitis Vinifera* (XP002285379.1), *Vitis quinquangularis* (ASR73556.1), *Rhodamnia argentea* (XP030523235.1), *Cephalotus follicularis* (GAV61182.1), *Canarium album* (QZM06937.1), *Mangifera indica* (XP044479746.1), and *Pistacia vera* (XP031284307.1).

MEGA was used to discuss the phylogenetic relationship between TbUGGT protein sequences and the corresponding proteins in different species. The UGT amino acid sequences of 20 plants were downloaded from the GenBank database for a cluster analysis (Figure 6). Higher scores indicated a closer relationship (the maximum score was 100). The closest relationship occurred between *Trapa bispinosa* Roxb. and pomegranate.

### 2.3. Prokaryotic Expression of TbUGGT

**Figure 7:**
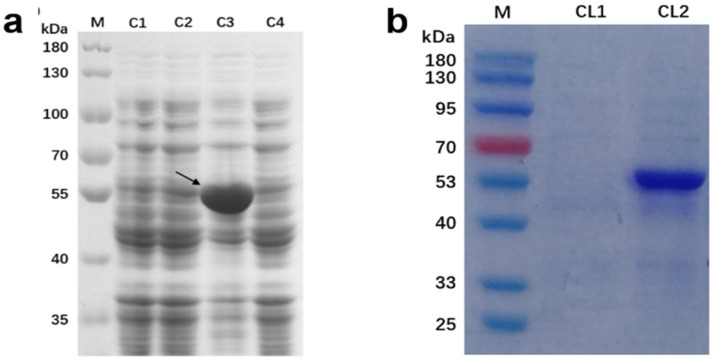
SDS-PAGE results of whole bacterial protein of recombinant strain containing pET-28a-TbUGGT (**a**) and SDS-PAGE results of purified protein (**b**). (**a**) Lane M, protein marker; Lane C1, non-induced whole bacterial protein containing recombinant plasmid; Lane C2, non-induced whole bacterial protein containing empty vector; Lane C3, whole bacterial protein containing recombinant plasmid after induction; Lane C4, whole bacterial protein containing empty carrier after induction. (**b**) Lane M, protein marker; Lane CL1, supernatant before bacterial fragmentation; Lane CL2, purified protein concentrate.

To obtain the recombinant expression strain, the recombinant plasmid PET-28a-Tbuggt was transformed into the *Escherichia coli* BL21(DE3) expression strain after colony PCR identification. IPTG was used as the inducer to induce fusion protein expression, and the bands were verified using SDS-PAGE electrophoresis (Figure 7a). Compared with the blank control group, specific protein bands of about 55 kDa (theoretically predicted value of 59.6 kDa) appeared in the experimental group, as indicated by the arrow in the figure.

Furthermore, the recombinant protein was purified with mass culture to eliminate the interference of other proteins, and the bacteria and the bacterial liquid were detected using SDS-PAGE (Figure 7b). The target protein band appeared in the bacterial lane at around 55 kDa, while the protein band did not appear in the bacterial liquid lane, indicating that the protein was expressed in *Escherichia coli*. However, the expressed proteins were mainly concentrated in the bacterial solution, and most of them existed in the form of inclusion bodies, which were broken to release the proteins. SDS-PAGE was used to detect the protein before and after purification (Figure 7b), and the target protein bands appeared at about 55 kDa, indicating that the protein was successfully expressed and purified in *Escherichia coli*.

### 2.4. Determination of Enzyme Activity of TbUGGT Protein In Vitro

**Figure 8:**
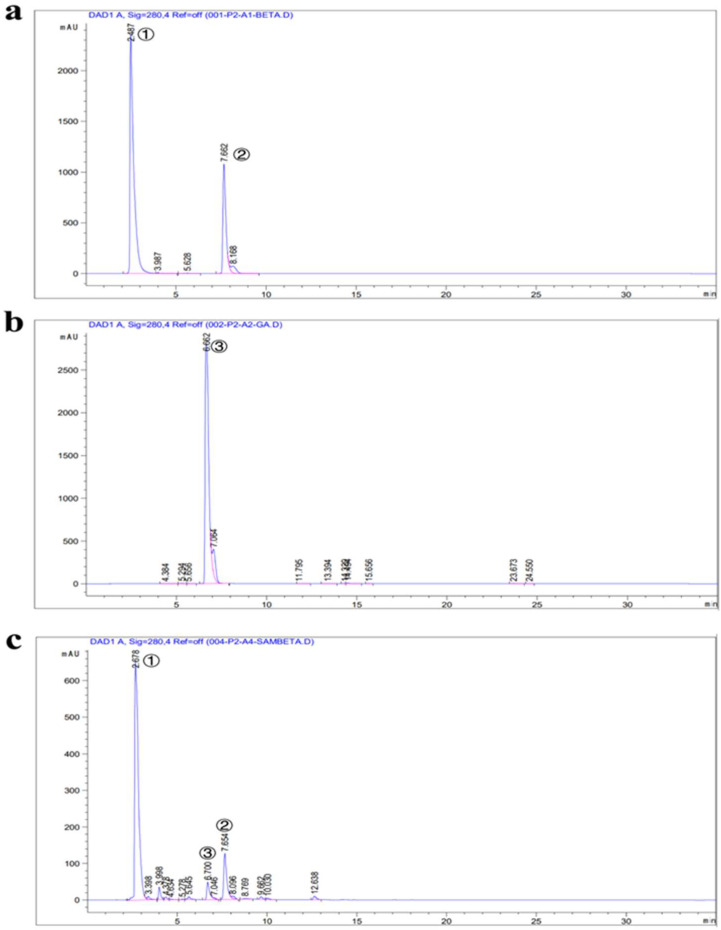
Determination of recombinant TbUGGT enzyme activity using HPLC. (**a**) UDPG(①) and GA(②) standard; (**b**) βG(③) standard; (**c**) experimental group.

**Figure 9:**
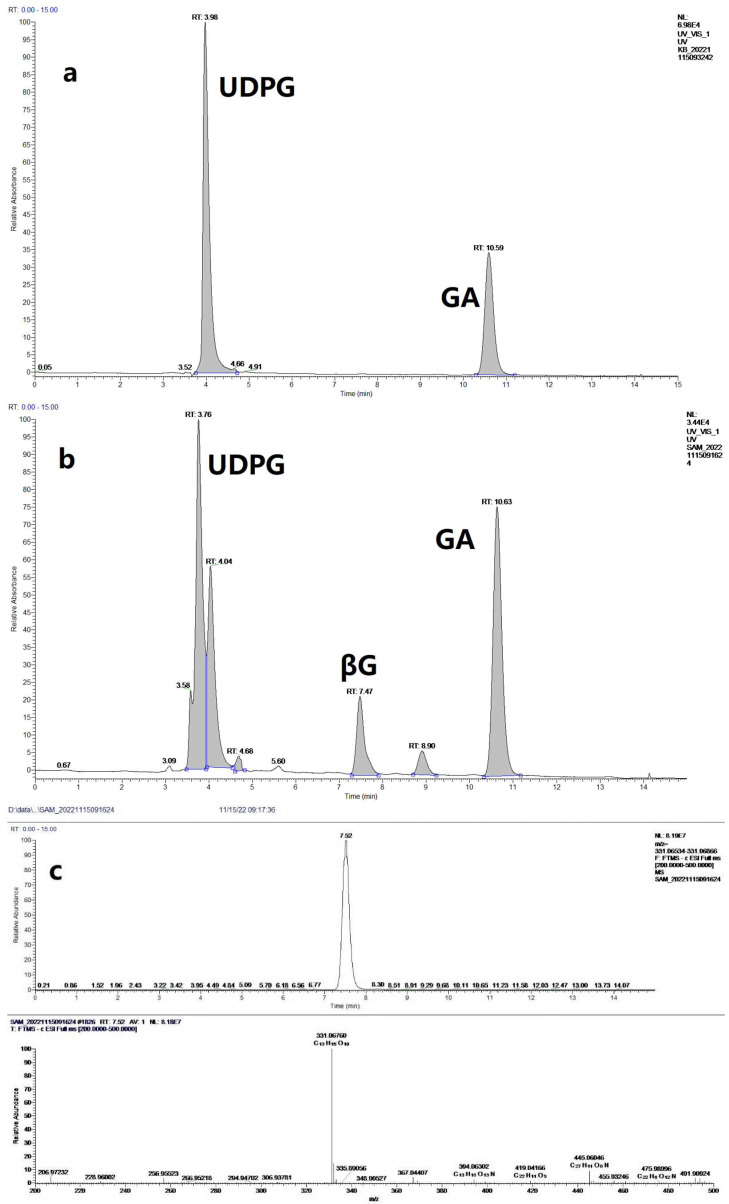
Determination of recombinant TbUGGT enzyme activity using LC-MS. (**a**) blank control group; (**b**) sample experiment group (**c**) βG mass spectrometry results.

The standard substances of βG, GA, and UDPG were detected under unified-liquid-phase conditions. The liquid-phase detection results (Figure 8) showed that the retention times of the three reference substances were 2.487 min for GA, 7.662 min for UDPG, and 6.662 min for βG. The experimental group showed a signal peak at 6.700, with a retention time similar to that of standard βG, indicating the successful production of βG. In order to confirm that the produced substance was indeed βG, LC-MS was used to verify the material composition of the sample and the blank control (Figure 9). The results showed contrast peaks at 7.28 min~7.90 min. There were βG characteristic ion fragments in the mass spectrum at 7.52 min *m*/*z* = 331.06760. The molecular formula was C_13_H_15_O_10_, and the molecular formula of βG is C_13_H_15_O_10_, which aligned with the negative ion scanning situation.

Therefore, it is speculated that the recombinant TbUGGT protein has some enzymatic activity and can catalyze the reaction between GA and UDPG to generate βG. However, the product peak area was small, and the conversion rate was low. Subsequent experiments may consider expanding the culture or increasing the amount of enzyme reaction to increase the yield.

## 3. Discussion

As an alien species, *Trapa bispinosa* Roxb. has been domesticated and cultivated in China [26]. Wuhan, China, is one of the cultivation bases of *Trapa bispinosa*, as the climate and environment are suitable for the growth and development of the plant [27]. Presently, research on *Trapa bispinosa* Roxb. focuses on the extraction of active ingredients [28,29], starch materials [30,31,32], pharmacological activity [33,34], etc. βG is one of the main active components of riboflavin and has significant medicinal value. Moreover, βG is the primary substrate of PGG, which has anti-cancer properties and has been extensively studied. However, the synthesis mechanism of *Trapa bispinosa* Roxb. remains unelucidated. Therefore, a series of experiments on βG biosynthesis were performed.

Many biochemical reactions are associated with glycosylation, and glycosyltransferase (GA) plays an essential role in plant growth and development, hormone balance, and toxic substance removal through glycosylation [35,36]. Meanwhile, a variety of glycosylation donors are involved in the glycosylation reaction. As a UDP-glycosylation donor-dependent enzyme, UGTs can selectively catalyze the site-directed glycosylation modification of natural and non-natural compounds. These are widely used in research and discovery of new drugs [37,38]. Here, the full-length gene of TbUGGT was amplified using a high-fidelity enzyme, and its amino acid sequence was compared with the glycosyltransferase of other species. The UGT family is highly conserved in different plant species [39]. The conserved functional domain allows the genes to maintain a certain similarity in catalytic potency. Currently, the UGT crystal structure has been obtained mainly in plants, such as cassava [40], Saffron [41], *Arabidopsis thaliana* [42,43], etc. These UGTs only recognize UDPG as a sugar donor [44]. In subsequent experiments in this study, TbUGGT selectively catalyzed UDPG and GA as substrates to generate βG, confirming the results of the functional analysis.

In this study, during the purification and expression of the TbUGGT protein, most proteins existed in the form of inclusion bodies, as predicted with ProtScale and signal peptide. The protein was a non-membrane and non-secretory protein. As reported in the literature, inclusion body proteins could not be inactivated after ultrasound [45]. Here, the TbUGGT protein was extracted and purified by referring to the particular extraction method of the *Escherichia coli* inclusion body [46]. *Escherichia coli* was used as host bacteria for heterologous expression, producing inclusion bodies and inhibiting protein expression, but its activity remained unaffected. The low content of late catalytic might have been related to the particular processing mode of proteins in *Escherichia coli*, resulting in the protein not being wholly purified [47,48]. The specific reasons need to be investigated in further studies.

The qRT-PCR results demonstrated that the expression levels in the shell and root were higher than those in the other two parts, which may have been related to the influence of phenolic tannins on plant growth. The higher shell expression may have been attributed to the accumulation of plant secondary metabolites in fruits. Studies have shown that the presence of binary phenol or polyphenol may inhibit the activity of indole acetate oxidase, reduce the degree of auxin oxidation, and promote plant growth [49]. Specific concentrations of plant endogenous phenols can enhance their rooting ability. As *Trapa bispinosa* Roxb. is a floating aquatic plant with many roots, the generation of polyphenols is essential to meet its rooting needs. Nevertheless, further research is required to elucidate the specific promoting mechanism.

## 4. Materials and Methods

### 4.1. Plant Materials

In this study, the plant materials of *Trapa bispinosa* Roxb. were collected in Jiangxia District, Wuhan City, Hubei Province (114.10° E, 30.27° N). After cleaning, the samples were treated with liquid nitrogen and immediately stored at −80 °C.

### 4.2. RNA Extraction and TbUGGT Enzyme Gene Cloning

RNA was extracted using The Plant Total RNA Isolation Kit (ENOVA BIO, Wuhan, China) The purity and concentration of RNA were determined using an ultra-micro spectrophotometer (MD2000D) and agarose gel electrophoresis (0.8% agarose). Single-strand cDNA was synthesized with PrimeScript IV 1st Strand cDNA Synthesis Mix (Takara Bio, Beijing, China). Specific primers were based on the TbUGGT sequence information obtained from *Trapa bispinosa* Roxb. transcriptome sequencing and designed using PremierX [24], as shown in Table 1. The TbUGGT enzyme gene was amplified using PCR with PrimeSTAR Max DNA Polymerase (Takara Bio, Beijing, China), and an OMEGA PCR purification kit was used to purify the amplified product. The size and quality of PCR products were determined with agarose gel electrophoresis (0.8% agarose).

### 4.3. TbUGGT Sequence Analysis and Phylogenetic Prediction

The physicochemical properties of the protein encoded by the TbUGGT gene were analyzed using ProtParam. Subsequently, conserved protein domains, their families, and functional sites were analyzed with CD Search, ScanProsite, and Pfam. Furthermore, ProtScale was used to analyze protein hydrophilicity, while TMHMM was used to predict the protein transmembrane helical region, and the protein signal peptide was predicted with SignalP. SOPMA was used to predict the protein’s secondary structure, and AlphaFold2 was used to construct the protein’s three-dimensional structure model [50]. The PDB file of the 3D structural model of the protein was downloaded, and the ligand molecules were downloaded from PubChem. Pymol-2.3.4 and AutoDockTools software applications were used to process the ligands and protein molecules, and Vina software was used for molecular docking. For visualization, the docking file was uploaded to Plip after PyMOL processing. The amino acid sequences of the encoded protein were compared using BLAST, and the homology was analyzed using DNAMAN. Mega-x was used to build the phylogenetic tree. Online website addresses are displayed in Table 2.

### 4.4. TbUGGT Prokaryotic Expression

(1) Vector construction and small-scale expression

The base sequence of the TbUGGT gene was optimized (GenScript Biotech Corp, Nanjing, China) according to the codon preference of *Escherichia coli*. Specific primers were designed, and restriction sites (EcoRI and NotI) were added, as shown in Table 1. The target gene was constructed in the PET-28a vector and transferred to DH5α. The plasmid was then extracted and sent for nucleic acid sequencing (Sangon Biotech (Shanghai) Co., Ltd., Shanghai, China). The corresponding colonies on the plate were carefully selected and inoculated into a kanamycin medium. The colonies were cultured overnight, and the plasmids were extracted and stored in glycerobacteria. The PET-28a recombinant vector was transformed into *Escherichia coli* BL21 (DE3), and colony PCR verification was performed using T7 primers, as shown in Table 2. BL21 was cultured in Luria-Bertani (LB) with kanamycin until the OD600 value reached about 0.6, and isopropyl -β-d-thiogalactoside (IPTG) was added at the final concentration of 0.5 mM to induce TbUGGT protein expression. Finally, 10% sodium dodecyl sulfate-polyacrylamide gel electrophoresis (SDS-PAGE) was performed to detect fusion protein expression.

(2) Expression and purification of large amounts of protein

Single colonies containing recombinant plasmids were selected and inoculated into a 10 mL LB liquid medium with corresponding resistance. After overnight culture in a 37 °C shaker, the colonies were transferred to 1 L of LB liquid medium and cultured until OD600 reached about 0.6 (duration of 2–3 h). The bacterial control solution was collected, and IPTG was added at a final concentration of 0.5 mM. After shaking the culture at 28 °C for 5 h, bacterial precipitates were collected using centrifugation (6000 RPM, 4 °C, 10 min), and the precipitates were cleaned twice with PBS to remove the residual medium. The bacterial precipitates were then collected and stored at −20 °C for future use.

The fusion protein was purified according to the instructions of the His-Tag Protein Purification Kit (Beyotime Biotechnology, Haimen, China). Subsequently, four milliliters of non-denatured lysate was added per gram of bacterial precipitate and complete suspension. The bacteria supernatant was collected using centrifugation. The BeyoGoldTM His-tag packaging column was prepared, and the upper cleaning column was loaded and washed 5 times with 1 mL of washing liquid; then, 0.5 mL of eluent was used ten times. The eluate of each tube was detected using SDS-PAGE electrophoresis, and the eluate that met the requirements was combined. The eluent was concentrated using an ultrafiltration tube, and SDS-PAGE was performed to detect 10 μL of the concentrated protein. The remaining concentrated solution was stored at −80 °C.

### 4.5. Enzyme Activity Detection of TbUGGT Protein

The total enzymatic reaction system was 100 μL, and 0.5 mM 3,4,5-trihydroxy benzoic acid (GA) and 2.5 mM uridine diphosphate glucose (UDPG) were added. Furthermore, 100 mM MES buffer containing 0.1% β-mercaptoethanol was used to provide a buffer environment. Next, the purified enzyme solution was added to the experimental group, while the enzyme solution was not added to the control group. After 3 h of reaction at 30 °C, methanol was added to terminate the reaction, and HPLC and LC-MS were used for detection. The liquid-phase conditions and methods are described below.

HPLC: chromatographic column, Agilent C18 column; mobile phase, 1% acetic acid water (A) and acetonitrile (B); injection volume, 20 μL; flow rate, 1.0 mL/min; column temperature, 35 °C; detection wavelength, 280 nm. Liquid-phase method: 0–10 min, 3–5% B; 10–15 min, 5–50% B; 15–25 min, 50–5% B; 25–30 min, 5–3% B; 30–35 min, 3% B. The liquid-phase diagram of standard βG was compared with the experimental results to confirm product formation.

LC-MS: chromatographic column, Waters ACQUITY C18 column (50 mm × 2.1 mm, 1.7 μm); mobile phase, 0.2% formic acid aqueous solution (A) and acetonitrile (B). Gradient elution: 0~1.5 min, 93% A; 1.5~8 min, 93%~80% A; 8~15 min, 80%~75% A. Volume flow rate, 0.4 mL/min; injection volume, 4 μL; column temperature, 35 °C.

Mass spectrometry conditions: negative ion scanning mode (ESI; *m*/*z* 100~1400); capillary voltage, 2.64 Kv; collision voltage, 45 V; drying gas temperature, 350 °C; source temperature, 150 °C; desolvent gas, N_2_, 800 L/Hr.

### 4.6. TbUGGT Expression Pattern

Four samples, including shell (FR), leaf (LR), stem (ST), and root (FR), were selected from the samples frozen at −80 °C, and the total RNA of the four samples was extracted using the Kit method. An ultra-micro spectrophotometer (MD2000D) and agarose gel electrophoresis (0.8% agarose) were used to evaluate the purity and concentration of RNA. Reverse transcription into cDNA was performed using the PrimeScriptTM RT Reagent Kit with gDNA Eraser (Takara Bio, Beijing, China) as the template for qRT-PCR. Table 1 displays the primer sequences (q-TbUGGT-F and q-TbUGGT-R) and reference gene EIF5A (C1168.2-F and C1168.2-R). The reaction systems were prepared according to TB Green^®^ Premix Ex TaqTM Ⅱ (Takara Bio, Beijing, China). The data were detected using CFX96TM real-time System (Bio-Rad, Wuhan, China) and analyzed using the 2-ΔΔCt method [51].

## 5. Conclusions

In this study, the gene TbUGGT was successfully cloned from *Trapa bispinosa* Roxb. After gene optimization, the nucleic acid and protein sequences were analyzed using bioinformatics and the phylogenetic tree and hosted into *Escherichia coli*. The gene was purified and successfully expressed in *Escherichia coli* BL12 (DE3). The HPLC results showed that TbUGGT could catalyze GA and UDPG to produce βG. This study found the catalytic role of TbUGGT in βG biosynthesis, laying the foundation for subsequent related studies on βG biosynthesis in *Escherichia coli*.

## Figures and Tables

**Table 1:** Primer sequences.

Primer Name	Sequence (5′-3′)
TbUGGT_F	CGGAATTCATGGGTTCCGAGTCCTCGC
TbUGGT_R	TAAAGCGGCCGCTCACGGGACCGGCTCTACC
TbUGGT_F	ggatccGAATTCATGGGAAGTGAATC
TbUGGT_R	ataagaatGCGGCCGCTTAGG
T7 F	TAATACGACTCACTATAGGG
T7 R	GCTAGTTATTGCTCAGCGG
q-TbUGGT_F	GTTTCAGATGGGAACGGCACTAGG
q-TbUGGT_R	TCTGCGATGCTGTGGGTTCAAAG
C1168.2 F	GCTTGAAGATATTGTCCCCTCATCCC
C1168.2 R	AGTCATCCTTTGTGCTGCCATTCTC

**Table 2:** Bioinformatic analysis tools.

Tool Name	Tool Web Site	Access Date
ProtParam	https://web.expasy.org/protparam/	12 February 2022
CD Search	https://www.ncbi.nlm.nih.gov/Structure/cdd/wrpsb.cgi?	12 February 2022
ScanProsite	https://prosite.expasy.org/scanprosite/	12 February 2022
Pfam	http://pfam.xfam.org/	12 February 2022
ProtScale	https://web.expasy.org/protscale/	12 February 2022
TMHHM	https://services.healthtech.dtu.dk/service.php?TMHMM	20 February 2022
SignalP	https://services.healthtech.dtu.dk/service.php?SignalP-6.0	20 February 2022
SOPMA	https://npsa-prabi.ibcp.fr/cgi-bin/npsa_automat.pl?page=npsa_sopma.htmL	30 July 2022
AphaFold2	https://github.com/lucidrains/alphafold2	5 November 2022

## Data Availability

Not applicable.

## Supplementary Materials

The following supporting information can be downloaded at: https://www.mdpi.com/article/10.3390/molecules27238374/s1, Figure S1: Domain prediction of TbUGGT protein (a–c), Figure S2: Hydrophilicity analysis of TbUGGT (a), Figure S3: Signal peptide prediction (a) and transmembrane prediction (b) of TbUGGT, Figure S4: Secondary structure prediction of TbUGGT.

## Sample Availability

Samples of *Trapa bispinosa* Roxb., vectors and strains carrying the TbUGGT gene, fermentation products of TbUGGT protein purification are available from the authors.
