# Supplementary figures and images for: Molecular Cloning, Expression, and Functional Analysis of Glycosyltransferase (TbUGGT) Gene from Trapa bispinosa Roxb
Source: Molecules. 2022 Nov 30;27(23):8374. doi: 10.3390/molecules27238374 (PMC9737334; doi:10.3390/molecules27238374)

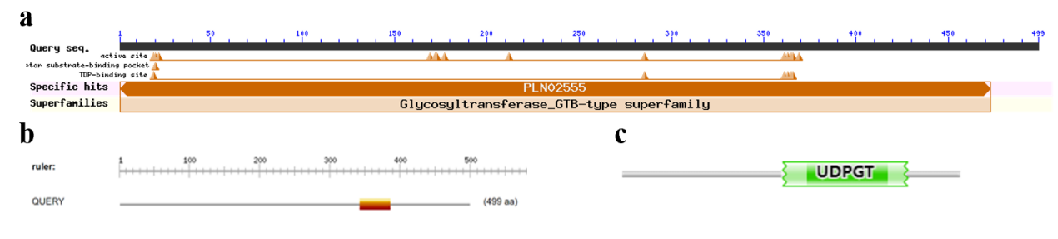

Supplement: Supplementary file 1 [file molecules-27-08374-s001.zip › Figure S1.png]

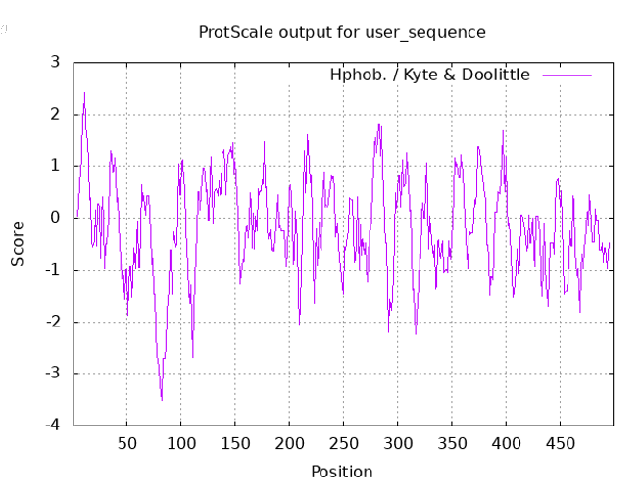

Supplement: Supplementary file 1 [file molecules-27-08374-s001.zip › Figure S2.png]

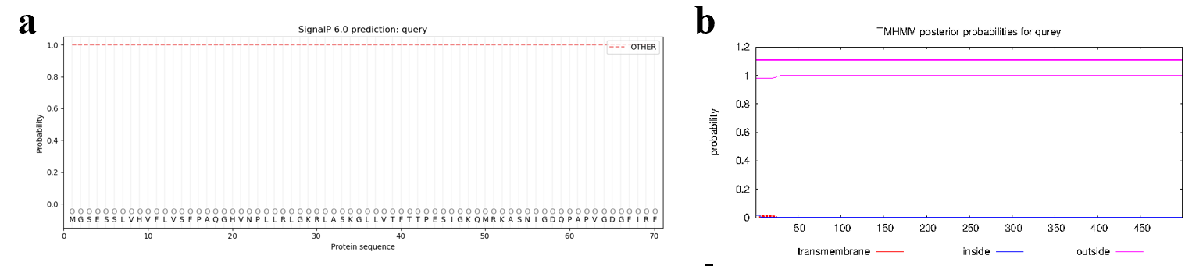

Supplement: Supplementary file 1 [file molecules-27-08374-s001.zip › Figure S3.png]

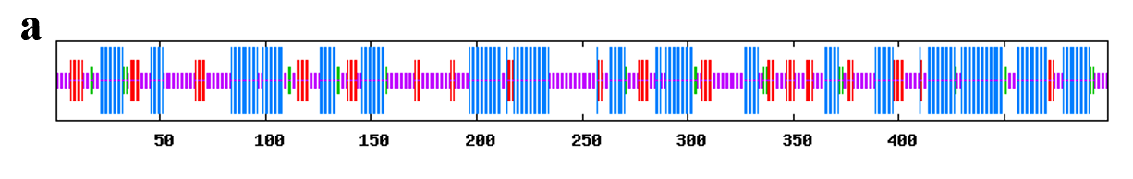

Supplement: Supplementary file 1 [file molecules-27-08374-s001.zip › Figure S4.png]

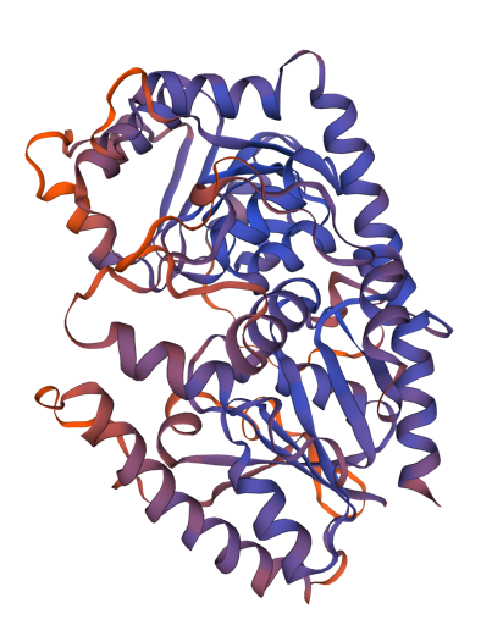

Supplement: Supplementary file 1 [file molecules-27-08374-s001.zip › Figure S5.png]

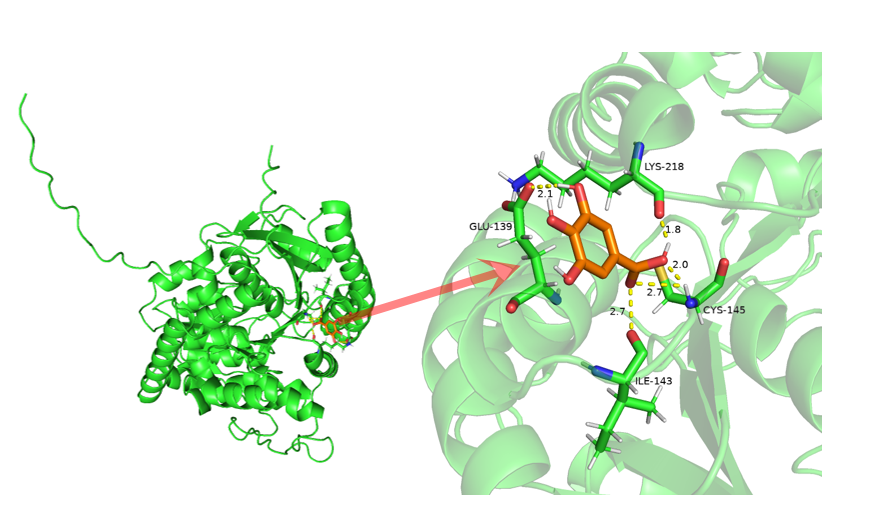

Supplement: Supplementary file 1 [file molecules-27-08374-s001.zip › Figure S6.png]
